# Investigational cyclin-dependent kinase 4/6 inhibitor GLR2007 demonstrates activity against isocitrate dehydrogenase wild-type glioblastoma and other solid tumors in mice xenograft models

**DOI:** 10.3389/fonc.2022.915862

**Published:** 2022-08-11

**Authors:** Lei Yin, Zhenglin Yao, Yue Wang, Michelle Mazuranic

**Affiliations:** ^1^ Gan & Lee Pharmaceuticals, Beijing, China; ^2^ Gan & Lee Pharmaceuticals USA Corp., Bridgewater, NJ, United States

**Keywords:** CDK4/6 inhibitors, blood–brain barrier penetration, breast cancer, colorectal cancer, IDH wild-type glioblastoma

## Abstract

Cyclin-dependent kinases, CDK4 and CDK6, are essential in regulating the cell cycle, which is disrupted in cancers like isocitrate dehydrogenase wild-type glioblastoma (GBM). Currently marketed CDK4/6 inhibitors, including abemaciclib, have shown preclinical efficacy in solid tumors, but factors such as poor blood–brain barrier (BBB) penetration limit their efficacy in GBM. GLR2007 is an investigational CDK4/6 inhibitor with the potential for improved BBB penetration. *In vitro* assays were used to assess the potency and inhibition of CDK4/6 enzymatic activity of GLR2007. Using *in vivo* assays, the distribution of radiolabeled GLR2007 in rats was determined through quantitative whole-body autoradiography. The antitumor efficacy of GLR2007 was evaluated in human GBM and breast cancer orthotopic mice xenograft models, and human lung, colorectal, and liver cancer in a subcutaneous xenograft model. In tumor cell line proliferation assays, GLR2007 inhibited proliferation at lower concentration values than abemaciclib in 19 of 20 GBM, five of seven breast, 20 of 21 lung, and 24 of 24 liver cancer cell lines. Total levels of radiolabeled GLR2007 in the brains of rats exceeded those in plasma by 2.3–4.5-fold from 2–6 hours after dosing. A xenograft model showed that, compared with vehicle control, 50 mg/kg GLR2007 induced 95.9% tumor growth inhibition (TGI) (*P*<0.001) in GBM orthotopic xenografts, 81.4% TGI (*P*=0.037) in breast cancer orthotopic xenografts, and 91.5% TGI (*P*<0.001) in colorectal cancer subcutaneous xenografts. These studies show possible BBB penetration of GLR2007 and demonstrate its potential as a CDK4/6 inhibitor for the treatment of solid tumors, including GBM.

## 1 Introduction

The cyclin-dependent kinases (CDKs) are a group of enzymes activated by forming heterodimeric complexes with cyclin proteins. Within the CDK group of proteins, CDK1, CDK2, CDK4, and CDK6 are involved in cell proliferation, regulating the transitions between different cell cycle phases (1). CDK4 and CDK6 share structural and biological properties, and are activated by D-type cyclins (CCNDs) in response to proliferative signaling. Activation of CDK4 and CDK6 leads to the phosphorylation and inactivation of retinoblastoma protein (pRb), thus mediating cell cycle transition from the G0/1 to the S phase (1–3). Dysregulation of the CCND–CDK4/6–cyclin-dependent kinase inhibitor 2A (CDKN2A)–Rb pathway may result in abnormal cell proliferation, which is a crucial mechanism of tumorigenesis in advanced solid tumor cancers, such as isocitrate dehydrogenase wild-type glioblastoma (GBM); hormone receptor (HR)-positive breast cancer; and non-small cell lung cancer (NSCLC) (2, 3). As CDK4 and CDK6 are crucial factors in tumor cell overproliferation, CDK4/6 inhibition is a potential therapeutic mechanism in these cancers (3). This potential was initially demonstrated through *in vitro* and *in vivo* studies, where CDK4/6 inhibitors were shown to cause cell cycle arrest, with an enhanced capacity to present antigens and stimulate cytotoxic T cells in breast cancer tumor cells (4).

Promising preclinical studies were followed by favorable clinical trial results, and four CDK4/6 inhibitors, palbociclib, abemaciclib, ribociclib, and trilaciclib are currently approved by the US Food and Drug Administration (FDA). The introduction of CDK4/6 inhibitors has been described as the most relevant advance in the management of HR-positive, Erb-B2 receptor tyrosine kinase 2 (ERBB2, formerly HER2)-negative metastatic breast cancer in recent years (5). Palbociclib, abemaciclib, and ribociclib are indicated for the treatment of HR-positive, ERBB2-negative breast cancer and are now considered a standard-of-care treatment in combination with an aromatase inhibitor or fulvestrant (6–9). Trilaciclib was recently approved to decrease the incidence of myelosuppression induced by some of the chemotherapy regimens used for the treatment of extensive-stage small-cell lung cancer (10).

Conversely, there remains an urgent need for additional therapeutic options in the treatment of the most common malignant primary brain tumor, GBM, which accounts for nearly half of all central nervous system (CNS) tumors (11). The 5-year survival rate of GBM beyond diagnosis is 7.2% and the median observed survival is 8 months, the lowest of all CNS tumors, including primary malignancies (11). There has been little improvement in patient survival rates over the last decade and limited progress in the approval of new therapies (12). The standard of care for newly diagnosed GBM, which has not changed substantially since 2005, remains as maximal surgical resection, followed by radiotherapy and chemotherapy with temozolomide (13–15).

A challenge to the development of effective targeted molecular therapies for GBM is the presence of the highly selective semipermeable blood–brain barrier (BBB), which prevents nearly all large molecules (>400 Da) and 98% of small-molecule drugs from entering the CNS (16, 17). Additionally, efflux transporters expressed on the luminal side of the BBB actively pump drugs that cross the plasma membrane back into the bloodstream, limiting the efficacy of even highly permeable drugs in the treatment of brain tumors (18). New therapies for GBM must be able to cross the BBB and attain therapeutic concentrations within the CNS, while limiting systemic exposure to the drug, so that patient safety is not compromised (19). CDK4/6 inhibitor therapy has potential for the treatment of GBM as the CCND–CDK4/6–CDKN2A–Rb pathway is one of the three pathways commonly disrupted in GBM, and in preclinical and phase 1 trials, palbociclib, abemaciclib, and ribociclib have been shown to cross the BBB at levels that would be expected to elicit kinase inhibition (20–22). However, to date, no clinical trials have demonstrated the efficacy of CDK4/6 inhibitors palbociclib and ribociclib in patients with GBM (22, 23). In a small cohort of GBM patients, abemaciclib was found in the cerebrospinal fluid and showed low therapeutic efficacy (23). GLR2007 is a novel investigational CDK4/6 inhibitor being developed for the potential treatment of advanced solid tumors, focusing on the treatment of GBM. The activity and efficacy of GLR2007 were demonstrated through preclinical *in vitro* assays, *in vivo* whole-body autoradiography, and *in vivo* xenograft models of GBM and other solid tumor cancers.

## 2 Materials and methods

### 2.1 *In vitro* assays of GLR2007 activity

#### 2.1.1 Enzymatic profiling assay

Inhibition of the catalytic activities of protein kinases, CDK4–cyclin D1 and CDK6–cyclin D1 (Invitrogen, Carlsbad, CA, USA), by GLR2007 (Gan & Lee Pharmaceuticals, Beijing, China) and palbociclib (Selleck Chemicals LLC, Houston, TX, USA) was assessed using a LANCE Ultra time-resolved fluorescence resonance energy transfer assay (PerkinElmer Inc., Waltham, MA, USA) according to the manufacturer’s instructions. GLR2007 was tested across a range of concentrations from 0.0017 nM to 100 nM. Palbociclib was tested across a range of concentrations from 0.0169 nM to 1000 nM. Results were calculated as half-maximal inhibitory concentration (IC_50_) and inhibition constant (*K*_i_). IC_50_ and *K*_i_ values were determined using the XLfit 205 model (ID Business Solutions Ltd, Guildford, UK) curve-fitting add-on for Microsoft Excel (Microsoft Corporation, Redmond, WA, USA).

#### 2.1.2 Cell proliferation assay

The effect of GLR2007 on cell proliferation was assessed in one mammary epithelial, 19 brain cancer, one neural, six breast cancer, 14 colon cancer, 29 liver cancer, and 21 lung cancer cell lines (**Supplementary Table 1**). To quantify cell proliferation, either the nuclei of live cells were counted following staining with 4’,6-diamidino-2-phenylindole (DAPI; Sigma-Aldrich, Burlington, MA, USA), or metabolically active cells were detected using a CellTiter-Glo assay (Promega Corporation, Madison, WI, USA). Cells from a range of human and mammalian tumor cell lines (American Type Culture Collection, Manassas, VA, USA) were seeded at 4000 cells per well in 100 μL of culture medium in a 96-well plate and incubated overnight at 37°C with 5% CO_2_. A range of concentrations of GLR2007 (0.01–10,000 nM), abemaciclib (1.5–10,000 nM; Gan & Lee Pharmaceuticals), or vehicle control dimethyl sulfoxide were added to each well at a volume of 20 μL, and cells were incubated at 37°C for 72 hours. In the DAPI staining method, cells were washed using 1× phosphate-buffered saline (PBS; Gibco Thermo Fisher Scientific, Waltham, MA, USA), fixed with 4% formaldehyde, permeabilized with 0.2% Triton X-100 (Sigma-Aldrich), washed again, and stained with 1 μg/mL DAPI to visualize cell nuclei. Cells were washed with 1× PBS three times, and 100 μL 1× PBS was added to each well. Plates were scanned using an IN Cell Analyzer 2200 (GE Healthcare Life Sciences, Chicago, IL, USA) and the number of cells per well was recorded. In the CellTiter-Glo assay, 60 μL of CellTiter-Glo luminescent solution was added to each well after incubation with the test compounds, and the assay plate was scanned using a Tecan Infinite M1000 PRO microplate reader (Tecan Group AG, Männedorf, Switzerland) to record luminescent signals. For both types of cell viability assay, dose-response curves for GLR2007 and abemaciclib were plotted, data fitted using a four-parameter logistic model in Prism 6.0 (GraphPad Software Inc., San Diego, CA, USA), and IC_50_ generated from each fitted curve.

### 2.2 *In vivo* assays of GLR2007 efficacy and tissue distribution

#### 2.2.1 Tissue distribution studies

To investigate the degree to which GLR2007 penetrated organs and tissues, and the levels of excretion of the compound, rats were dosed with radiolabeled GLR2007 and whole-body autoradiography was performed. Sprague Dawley rats (Vital River Laboratory Animal Technology Co., Beijing, China; N=38, female n=19; 8–11 weeks old) were maintained in humidity- and temperature-controlled conditions on a 12-hour light/dark cycle, with food (certified rat diet; Jiangsu Xietong Pharmaceutical Biological Engineering Co., Ltd, Jiangsu, China) and water available *ad libitum*. Rats were administered a single oral dose of 6 mg/100 μCi/kg of [^14^C]GLR2007. Urine, feces, and cage rinse/wash up to 168 hours after treatment were collected from six intact rats (three male, three female); bile, urine, feces, and cage rinse/wash up to 72 hours after treatment were collected from eight bile duct cannulated (BDC) rats (four male, four female). Heart, lung, liver, spleen, kidneys, whole brain, stomach tract wall, intestinal tract wall, skeletal muscle, body fat, reproductive organs (uterus and ovary for female rats, testis and epididymis for male rats) were collected at 2, 6, 24, and 72 hours after treatment (three male, three female per time point). The total radioactivity (TRA) of plasma, bile, urine, and cage rinse/wash samples was directly analyzed by liquid scintillation counting. Fecal and tissue homogenates and blood were combusted using a PerkinElmer Oxidizer (PerkinElmer Inc.), and the generated [^14^C]CO_2_ was captured in ^14^C scintillation cocktail (Shimadzu Corporation, Kyoto, Japan), followed by analysis using a PerkinElmer Liquid Scintillation Counter (PerkinElmer Inc.). The TRA levels determined in bile, urine, feces, and cage rinse/wash were used to calculate the percentage of the administered dose excreted. Mean TRA values of whole brain and plasma at 2, 6, 24, and 72 hours were used to calculate the area under concentration–time curve from time zero to 72 hours (AUC_0–72_), using a noncompartmental model in Phoenix WinNonlin v6.3 software (Certara, Princeton, NJ, USA).

#### 2.2.2 Tumor xenograft studies

The efficacy of GLR2007 was evaluated in human GBM and breast cancer orthotopic cell-line-derived xenografts in NOD/SCID mice (Shanghai Lingchang Biotechnology Co. Ltd, Shanghai, China) and BALB/c nude mice (Shanghai Sino-British SIPPR/BK Laboratory Animal Co. Ltd, Shanghai, China), and human lung, colorectal, and liver cancer subcutaneous cell-line-derived xenografts in BALB/c nude mice (Nanjing University National Resource Center, Nanjing, China, or Beijing Anikeeper Biotech Co., Beijing, China). Mice were maintained in humidity- and temperature-controlled conditions with food and water available *ad libitum*.

In a GBM orthotopic xenograft study, female BALB/c nude mice were inoculated intracranially at 6–8 weeks old with 3×10^5^ U87-luc tumor cells plus Matrigel (Corning Life Sciences, Tewksbury, MA, USA) in a 4:1 ratio in PBS at the right frontal lobe. Tumor growth was quantified using intraperitoneal administration of 150 mg/kg luciferin to anesthetized mice, and tumor bioluminescence was measured using an IVIS Lumina II imaging system (PerkinElmer Inc.). In one study, mice were randomized at day 6 after tumor implantation (mean bioluminescence of 2.81×10^7^ photons/sec), and in the other, mice were randomized at day 14 or day 15 (mean bioluminescence of 7.49×10^6^ photons/sec).

In two breast cancer orthotopic xenograft models, female NOD/SCID mice were subcutaneously implanted with an estrogen pellet (17β-estradiol; Innovative Research of America, Sarasota, FL, USA) one day before tumor inoculation. Mice were inoculated at 6–7 weeks old with 2×10^7^ MCF-7 tumor cells plus Matrigel in a 1:1 ratio in PBS at a mammary fat pad or in the right flank. Randomization was performed when the mean tumor volume reached 135–138 mm^3^.

For subcutaneous models, female BALB/c nude mice were inoculated at 4–8 weeks old with 3×10^6^–1×10^7^ tumor cells (see **Table 1** for xenograft model cell types) in the right flank for tumor development. Randomization was performed when the mean tumor volume reached 118–171 mm^3^.

**Table 1:** Tumor growth inhibition and changes in median survival time in mouse xenograft models.

Xenograft model	Treatment	Number of mice	Dose (mg/kg)	Length of dosage (days)	Tumor growth measurement – days since inoculation	TGI (%)^a^	TGI *P* value	Increase in median survival time (%)^b^	Survival time *P* value
**Glioblastoma**									
Subcutaneous BN2289	GLR2007	8	5	28	21	4.9	<0.001	–	–
	GLR2007	8	25			39.4	<0.001	–	–
	GLR2007	8	50			56.4	<0.001	–	–
	Abemaciclib^c^	8	25			24.9	<0.001	–	–
	Palbociclib^c^	8	25			34.0	<0.001	–	–
Orthotopic U87-luc	GLR2007	8	12.5	42	21	86.3	0.588	50.0	0.0009
	GLR2007	8	50			95.9	0.496	182.6	<0.0001
	Abemaciclib	8	150			79.0	0.666	54.4	0.0002
**Breast cancer**									
Orthotopic MCF-7	GLR2007	8	10	21	21	43.5	0.033	–	–
	GLR2007	8	25			37.4	0.017	–	–
	GLR2007	8	50			49.6	0.001	–	–
	Abemaciclib^c^	8	25			33.0	0.154	–	–
	Palbociclib^c^	8	25			45.5	0.057	–	–
Orthotopic MCF-7	GLR2007	10	25	28	25	61.4	0.137	–	–
	GLR2007	10	50			81.4	0.037	–	–
	Abemaciclib^c^	10	25			48.6	0.222	–	–
	Palbociclib^c^	10	25			77.7	0.045	–	–
**Non-small cell lung cancer**									
Subcutaneous NCI-H1975	GLR2007	8	10	22	16	27.6	0.025	–	–
	GLR2007	8	25			50.0	<0.001	–	–
	GLR2007	8	50			68.9	<0.001	–	–
	Abemaciclib^c^	8	25			6.9	0.911	–	–
	Palbociclib^c^	8	25			33.6	0.002	–	–
Subcutaneous NCI-H2228	GLR2007	10	50	28	34	33.9	0.003	–	–
**Colorectal cancer**									
Subcutaneous COLO 205	GLR2007	10	5	26	25	42.6	0.003	–	–
	GLR2007	10	25			72.2	<0.001	–	–
	GLR2007	10	50			91.5	<0.001	–	–
	Abemaciclib^c^	10	25			57.7	<0.001	–	–
	Palbociclib^c^	10	25			52.9	<0.001	–	–
**Liver cancer**									
Subcutaneous LI1088	GLR2007	8	25	28	25	57.7	0.038	–	–
	GLR2007	8	50			77.7	0.003	–	–
	Abemaciclib^c^	8	25			22.4	0.578	–	–
	Palbociclib^c^	8	25			16.4	0.802	–	–

a Calculated as change in mean tumor volume from baseline for treatment group, as a percentage of change in vehicle group.

b Calculated as percentage increase in median survival time of treatment group versus vehicle group.

c Palbociclib and abemaciclib were tested only at the doses stated.

TGI, tumor growth inhibition.

Upon randomization, mice were administered with single daily doses of GLR2007, abemaciclib (Gan & Lee Pharmaceuticals), or palbociclib (Gan & Lee Pharmaceuticals) in vehicle control, or vehicle alone, by oral gavage (see **Table 1** for doses and days of treatment). Treatment efficacy was evaluated by determining relative tumor growth inhibition (TGI), calculated as: (delta [vehicle group] – delta [treatment group])/delta [vehicle group] × 100%.

#### 2.2.3 Statistical analysis

Statistical analysis of differences in mean tumor volume among groups was conducted *via* an independent samples *t*-test or one-way analysis of variance (ANOVA; two-sided Dunnett’s test). For comparison among three or more groups, a one-way ANOVA was performed. Multiple comparison procedures were applied after ANOVA when significant F-statistics (ratio of treatment variance to error variance) were obtained. Comparisons between groups were carried out using the Games test. *P*<0.05 was considered to be statistically significant. Survival times were analyzed using the Kaplan–Meier method, with curves constructed for each group and the log-rank (Mantel–Cox) test used to compare survival curves between groups. Body weight change was calculated based on the weight on the first day of dosing.

## 3 Results

### 3.1 Enzymatic activity and cell proliferation

**Figure 1:**
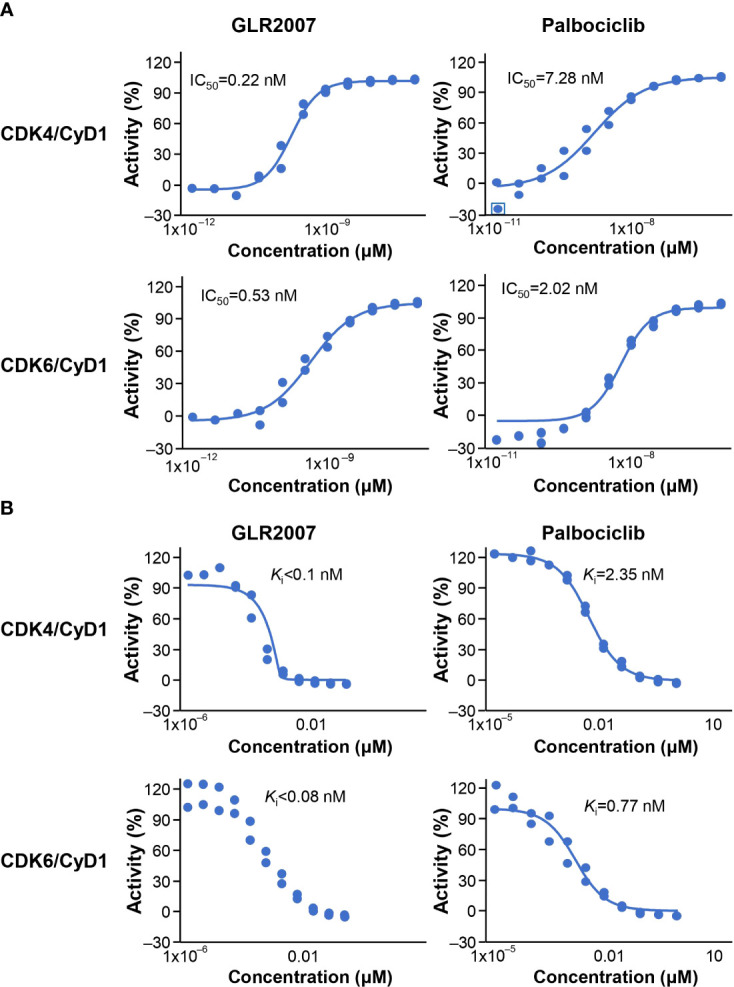
Inhibition of CDK4/CycD1 and CDK6/CyD1 enzymatic activity by GLR2007 and palbociclib. Results were calculated across 11 concentrations, measured in duplicates, within the range of 0.0017–100 nM GLR2007 and 0.0169–1000 nM palbociclib, and inhibition of kinase activity expressed as **(A)** IC_50_ and **(B)** *K*_i_. Mean inhibition values are shown within each plot. CDK, cyclin-dependent kinase; CyD1, cyclin D1; IC_50_, half-maximal inhibitory concentration; *K*_i_, inhibition constant.

In CDK4 and CDK6 enzymatic activity assays, GLR2007 showed greater inhibition than palbociclib in IC_50_ (**Figure 1A**) and *K*_i_ (**Figure 1B**) dose-response curves. More potent inhibition of CDK4 enzymatic activity was exhibited by GLR2007, compared with palbociclib (IC_50_ 0.22 nM versus 7.28 nM; *K*_i_ *<*0.1 nM versus 2.35 nM). The IC_50_ value of GLR2007 showed a 33.1-fold difference, and the *K*_i_ value showed a 23.5-fold difference compared with these values in palbociclib. GLR2007 also demonstrated more potent inhibition of CDK6 enzymatic activity, compared with palbociclib (IC_50_ 0.53 nM versus 2.02 nM; *K*_i_ *<*0.08 nM versus 0.77 nM), with the IC_50_ value of GLR2007 showing a 3.8-fold difference, and the *K*_i_ value a ~9.6-fold difference compared with these values in palbociclib.

For cell proliferation assays, cell viability data following treatment with GLR2007 were obtained for 90 cell lines, and of these, data following treatment with abemaciclib were obtained for 86 cell lines (**Supplementary Table 1**). GLR2007 inhibited cell proliferation to an equal or greater extent than abemaciclib in 77 of 86 cell lines (89.5%) (IC_50_ fold-difference range = 0.03–6.77; median = 0.42; mean ± standard deviation (SD) = 0.70 ± 1.06). In blastoma cell lines, GLR2007 inhibited cell proliferation to an equal or greater extent than abemaciclib in 19 of 20 cell lines (95%) (IC_50_ fold difference range = 0.15–3.73; median = 0.47; mean ± standard deviation (SD) = 0.65 ± 0.76). In the non-malignant MCF-10A mammary epithelial cell line, GLR2007 demonstrated less cell proliferation inhibition than abemaciclib (IC_50_ 2.73 µM versus 1.77 µM, respectively).

### 3.2 Tissue distribution and BBB penetration

**Figure 2:**
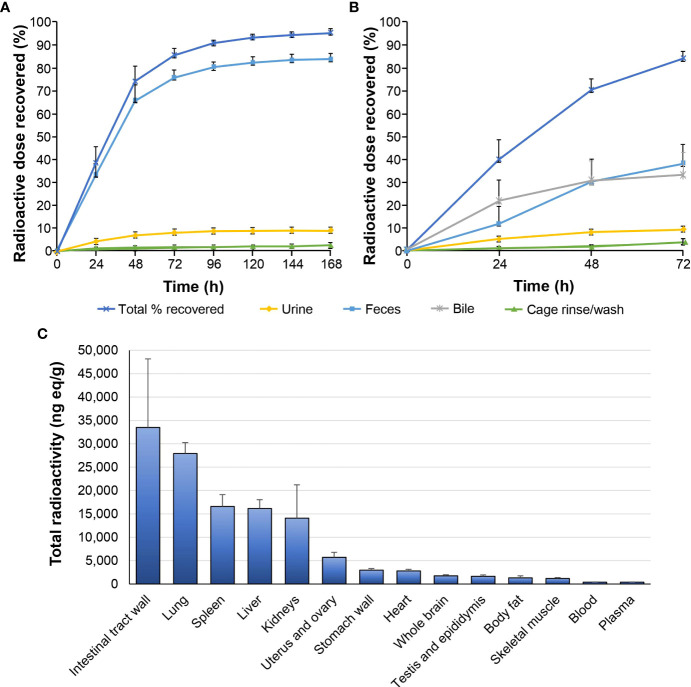
Tissue distribution and excretion of GLR2007 in Sprague Dawley rats. Intact and bile duct cannulated Sprague Dawley rats were administered with a single oral dose of [^14^C]GLR2007 at 6 mg/100 μCi/kg. **(A)** Cumulative excretion of radioactivity in intact rats over 168 hours following [^14^C]GLR2007 dosing. **(B)** Cumulative excretion of radioactivity in bile duct cannulated rats over 72 hours following [^14^C]GLR2007 dosing. **(C)** Total radioactivity (ng eq/g, mean ± standard deviation) detected in blood, plasma, and tissue of intact rats at 6 hours following [^14^C]GLR2007 dosing.

Following a single 6 mg/100 μCi/kg oral dose of [^14^C]GLR2007 administered to intact rats, 95.5% of the dose was recovered within 168 hours after treatment, and derived radioactivity was mainly excreted *via* feces (84.10%) (**Figure 2A**). Most (74.3%) of the [^14^C]GLR2007 was recovered within the first 48 hours after treatment. Following a single 6 mg/100 μCi/kg oral dose of [^14^C]GLR2007 to BDC rats, 84.2% of the dose was recovered within 72 hours after dosing, with 33.3% in bile (**Figure 2B**). Based on the percentage of the original dose of [^14^C]GLR2007 that was excreted in bile and urine from BDC rats, it is predicted that at least 42.4% of a dose of GLR2007 will be absorbed. After 6 hours from dosing, radioactivity in intact rats was rapidly distributed throughout the body *via* the blood and was mainly detected in the intestinal tract wall, lungs, spleen, liver, and kidneys (**Figure 2C**). Total radioactivity levels detected in whole-brain tissue exceeded those in plasma by 2.31-fold from 2 hours after dosing ([mean ± SD] whole brain = 497 ± 109 ng eq/g; plasma = 215 ± 48.9 ng eq/g). The maximum concentration (C_max_) of radioactivity in blood, plasma, and all tested tissue was observed at 6 hours after treatment, except the stomach wall, in which C_max_ was attained at 2 hours, and testis, epididymis, and kidney, which peaked at 24 hours. In whole-brain tissue, the C_max_ value (1774 ± 239 ng eq/g) was 4.51-fold higher than in plasma (393 ± 46.1 ng eq/g) (**Table 2**). In female rats, the AUC_0–72 h_ of whole-brain tissue (52,302 h·ng eq/g) was 3.97-fold higher than in plasma (13,165 h·ng eq/g), and in male rats, the AUC_0–72 h_ of whole-brain tissue (33,554 h·ng eq/g) was 4.09-fold higher than in plasma (8213 h·ng eq/g).

**Table 2:** Total detected radioactivity in blood, plasma, and tissue following a single 6 mg/100 μCi/kg oral dose of [^14^C]GLR2007 to Sprague Dawley rats (mean ± standard deviation, n=6 per time point).

	Total detected radioactivity (ng eq/g)			
Tissue	2 hours	6 hours	24 hours	72 hours
Intestinal tract wall	27,015 ± 7125	33,495 ± 14,685	8760 ± 3716	850 ± 407
Lung	17,352 ± 15,323	27,919 ± 2346	12,148 ± 4665	1708 ± 804
Kidneys	7443 ± 3725	14,089 ± 7132	17,410 ± 12,359	2862 ± 1288
Spleen	5857 ± 1380	16,613 ± 2520	13,285 ± 6920	2149 ± 1203
Liver	12,563 ± 1168	16,169 ± 1884	5451 ± 1887	1598 ± 752
Uterus and ovaries	1518 ± 922	5710 ± 1098	5443 ± 1242	1028 ± 169
Stomach tract wall	8015 ± 2054	2969 ± 358	1145 ± 344	268 ± 91.2
Heart	1323 ± 213	2815 ± 346	1705 ± 650	524 ± 202
Whole brain	497 ± 109	1774 ± 239	792 ± 341	102 ± 61.5
Testes and epididymis	315 ± 36.1	1649 ± 330	2139 ± 474	392 ± 76.6
Body fat	638 ± 222	1318 ± 459	474 ± 152	125 ± 36.5
Skeletal muscle	370 ± 124	1198 ± 177	530 ± 217	105 ± 41.3
Blood	211 ± 46.9	399 ± 46.6	190 ± 92.2	BLQ
Plasma	215 ± 48.9	393 ± 46.1	188 ± 92.9	34.9 ± 11.6

BLQ, below the limit of quantitation.

### 3.3 Antitumor efficacy in xenograft models

#### 3.3.1 Glioblastoma xenograft model

**Figure 3:**
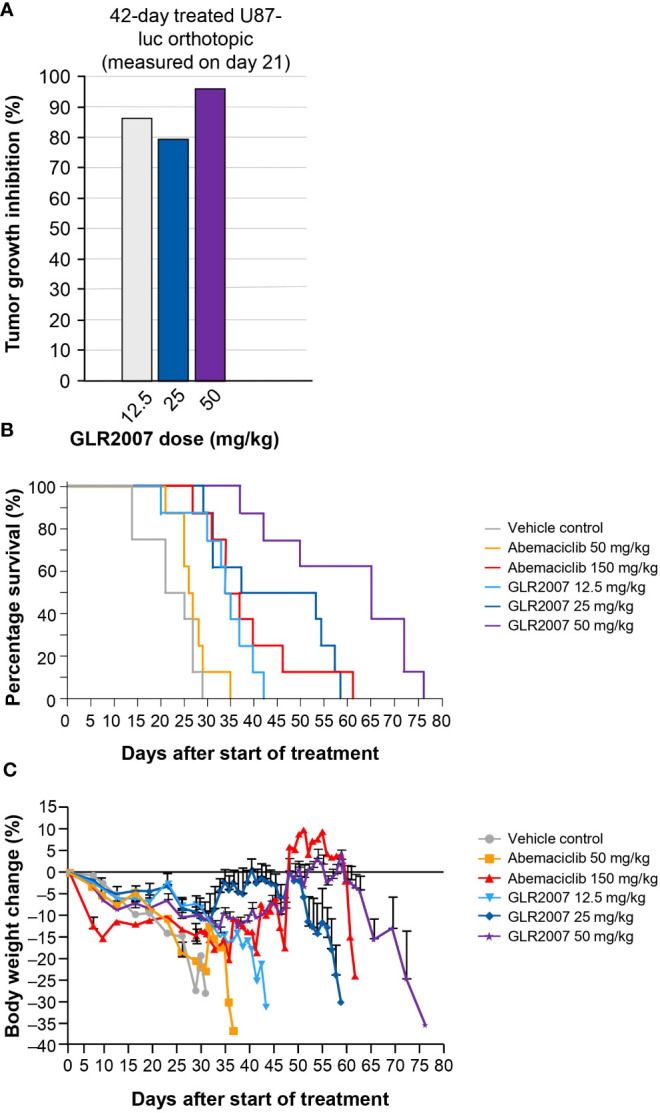
Tumor growth inhibition, survival rates and body weight changes in a mouse GBM xenograft model following treatment with a range of doses of GLR2007 or abemaciclib. **(A)** Tumor growth inhibition in a mouse orthotopic GBM xenograft model following 42 days of treatment with a range of doses of GLR2007, administered once daily, compared with vehicle control. The tumor growth was measured on day 21. **(B)** Kaplan–Meier survival curves of 42-day treated orthotopic U87-luc xenograft mice following treatment with a range of doses of GLR2007 or abemaciclib. **(C)** Relative body weight changes of orthotopic U87-luc xenograft mice treated for 42 days with a range of doses of GLR2007 or abemaciclib. Error bars represent the error of the mean. GBM, glioblastoma.

**Figure 4:**
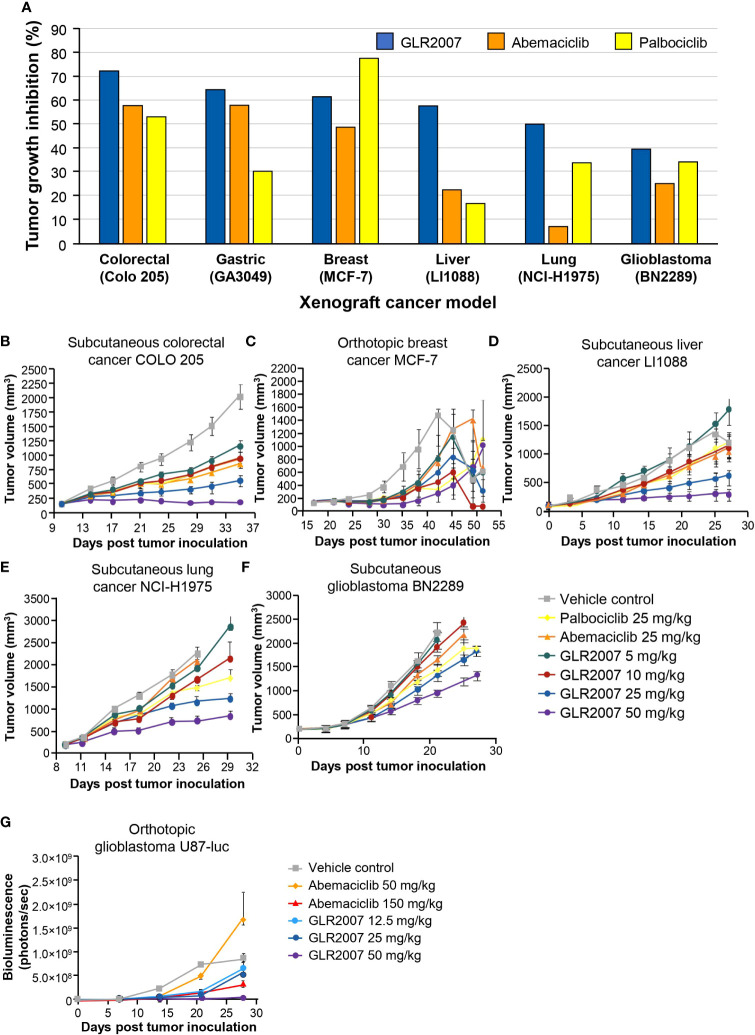
Tumor growth inhibition in mouse xenograft models treated with GLR2007, abemaciclib, and palbociclib. **(A)** Tumor growth inhibition in mouse xenograft cancer models following treatment with 25 mg/kg body weight of GLR2007, abemaciclib, and palbociclib, compared with vehicle control. **(B–F)** Tumor growth curves of mouse xenograft cancer models. **(B)** Subcutaneous colorectal cancer COLO 205, treated for 26 days, measured on day 25. **(C)** Orthotopic breast cancer MCF-7, treated for 28 days, measured on day 25. **(D)** Subcutaneous liver cancer LI1088, treated for 28 days, measured on day 25. **(E)** Subcutaneous lung cancer NCI-H1975, treated for 26 days, measured on day 16. **(F)** Subcutaneous glioblastoma BN2289, treated for 22–28 days, measured on day 21. **(G)** Tumor growth curve reflected by bioluminescence density of orthotopic glioblastoma U87-luc, treated for 42 days with a range of doses of GLR2007 or abemaciclib.

In orthotopic GBM U87-luc xenografts treated for 42 days, measured on day 21, a 50 mg/kg dose of GLR2007 resulted in TGI of 95.9%; however, the death of two animals in the vehicle group and one in the 12.5 mg/kg GLR2007 group before final measurements reduced statistical power, resulting in nonsignificant *P* values for TGI in this experiment (*P*=0.496) (**Figure 3A**; **Table 1**). In the U87-luc orthotopic xenograft model, median survival time was 182.6% (*P*<0.0001) longer in mice treated with 50 mg/kg GLR2007, compared with vehicle controls (**Figure 3B**; **Table 1**). Weight loss was comparable between the GLR2007 and abemaciclib groups (**Figure 3C** **).** At equivalent doses (25 mg/kg), GLR2007 demonstrated noninferior TGI to abemaciclib and palbociclib in glioblastoma xenograft models (**Figures 4A, F, G** **;** **Table 1**).

#### 3.3.2 Other solid cancer xenograft models

Across a range of orthotopic and subcutaneous tumor cell xenografts, GLR2007 showed significant TGI versus vehicle control, with numerically greater inhibition than abemaciclib and palbociclib in most models (**Table 1**). GLR2007 induced TGI of up to 81.4% (*P*=0.037) in MCF-7 breast cancer xenografts, up to 68.9% (*P*<0.001) in NCI-H1975 NSCLC xenografts, up to 91.5% (*P*<0.001) in COLO 205 colorectal cancer xenografts, and up to 77.7% (*P*=0.003) in LI1088 liver cancer xenografts, compared with vehicle controls. At equivalent doses (25 mg/kg), GLR2007 demonstrated numerically greater TGI over abemaciclib and palbociclib in lung, colorectal, and liver cancer xenograft models, and over abemaciclib in breast cancer xenograft models (**Figures 4A–E**).

## 5 Discussion

CDK4 and CDK6 are targets for cancer therapies because they are part of the CCND–CDK4/6–CDKN2A–Rb pathway, which mediates the cell cycle transition from G0/1 to S phase. This pathway is frequently dysregulated in cancers, leading to the over proliferation of cells (3). This study demonstrated the activity and efficacy of a novel investigational CDK4/6 inhibitor GLR2007 in preclinical models of solid tumors including GBM, and lung, liver, breast, and colorectal cancers. Treatment with GLR2007 inhibited cell proliferation to an extent equivalent to or greater than CDK4/6 inhibitor abemaciclib in 77 (89.5%) of the 86 mammalian tumor cell lines tested (**Supplementary Table 1**). GLR2007 exhibited less toxicity than abemaciclib on cell proliferation in the human mammary epithelial cell line (**Supplementary Table 1**). GLR2007 was also shown to inhibit the enzymatic activity of CDK4 and CDK6, and induce G1 arrest in cell line U87-MG, a model of GBM brain cancer (19).

GBM has the highest malignancy and fatality rates of all brain cancers, but there have been no major advances in the standard of care for GBM in over 15 years (14, 24). The biological characteristics of brain tumors, the unique microenvironment of the brain, and the presence of the BBB combine to cause brain tumors to be resistant to many treatments (25). To improve patient outcomes, there is a need for new GBM therapies, which must be able to cross the BBB and work efficaciously within the brain (14, 24).

In previous preclinical studies of CNS penetration, palbociclib’s unbound brain-to-plasma partition coefficient (Kp,uu) was 0.01 within 5 minutes after intravenous administration of 1 mg/kg (21, 26). Abemaciclib had a Kp,uu value of 0.03 in mice after an oral dose of 30 mg/kg, indicative of active efflux (21). Using cerebral microdialysis, the Kp,uu value for ribociclib was 0.105 in CD1 nude mice after oral administration of 100 mg/kg (27). In contrast, compound GLR2007 exhibited a more significant penetration of the BBB with a total brain/plasma ratio value of 4.10 and the Kp,uu value of 0.23 in mice after an oral dose of 10 mg/kg. It was also demonstrated that compound GLR2007 is not a substrate of efflux transporters P-glycoprotein and breast cancer resistance protein (BCRP) in our previous publication (19). Our earlier data combined with this study, the radioactivity level of GLR2007 in the brain tissue of treated rats was 2.31-fold greater than in plasma at 2 hours after treatment (**Table 2**) and the AUC_0–72 h_ of brain tissue was up to 4.09-fold higher than that of plasma, demonstrates substantially greater CNS penetration by GLR2007 compared with that previously reported for other marketed CDK4/6 inhibitors (21, 27).

Abemaciclib has previously demonstrated antitumor efficacy in orthotopic U87-MG GBM rat xenografts, increasing survival time by 37.4% compared to vehicle control (21). In these studies, GLR2007 was shown to increase survival time by up to 182.6% and inhibit tumor growth by up to 95.9% in orthotopic GBM mouse xenografts. Weight loss curves, which reflect weight loss due to toxicity of the drug and the disease, showed noninferior changes in mice treated with GLR2007 compared with abemaciclib. Efficacy of GLR2007 was also demonstrated in breast cancer and lung cancer mouse xenograft models, with tumor inhibition of up to 81.4% and 68.9%, respectively – numerically higher than the level of inhibition observed following treatment with palbociclib and abemaciclib (**Table 1**).

In conclusion, these studies suggest that GLR2007 may be as efficacious in the treatment of GBM and other advanced solid tumor cancers as the approved CDK4/6 inhibitors, supporting further investigation of GLR2007 through clinical trials. There is an ongoing debate about whether abemaciclib and palbociclib can penetrate the BBB. Previous preclinical studies have shown that abemaciclib and palbociclib penetrate the BBB and reach concentration levels in rodent brains that would be expected to inhibit the activity of CDK4/6 (21–23). However, it has been suggested that this might be because previously shown BBB penetration was obtained in models less relevant to GBM (28). Furthermore, Patnaik et al. demonstrate a low therapeutic effect of abemaciclib in patients with GBM; however, the small cohort of patients (n=17) warrants further investigation (23). Randomized clinical trials are currently underway to evaluate palbociclib, abemaciclib, ribociclib, and trilaciclib in solid tumor cancers, including GBM, NSCLC, ovarian cancer, and colorectal cancer, both as monotherapies and in combination with signaling pathway inhibitors (29). A phase 1b/2 open-label multicenter clinical trial is currently recruiting to establish the safety, tolerability, and optimal dosing strategy of GLR2007 in subjects with NSCLC, brain metastases of breast or NSCLC origin, or GBM (NCT04444427). In addition, GLR2007 has been granted orphan drug designation by both the FDA and the European Medicines Agency for the treatment of malignant glioma (30, 31).

The activity and efficacy of GLR2007 were established in tumor cell lines and mouse xenograft models (human GBM and breast, brain, lung, colorectal, and liver cancer). Moreover, although the relative distribution of GLR2007 to the brain is low compared to other tissues, this study shows the potential of BBB penetration and delivery to the CNS. These preclinical studies demonstrate the prospect of this novel investigational CDK4/6 inhibitor for the treatment of GBM and other advanced solid tumors. Ongoing clinical trials will establish whether these preclinical findings translate to clinical benefits for patients.

This study used the U87 GBM model, which has limitations regarding its suitability as a preclinical model of recurrent high-grade glioma. There are concerns that the U87 mouse model might not recapitulate the highly infiltrative growth of glioma cells accurately, and that the highly permeable vasculature is not ideal (32). Despite this, GLR2007 shows similar efficacy as abemaciclib (**Figures 3B** and **4G**). Moreover, the subcutaneous PDX GBM model, BN2289 (**Table 1**) also showed similar efficacy between GLR2007 and abemaciclib. Therefore, we conclude that these data together with the BBB penetration (**Table 2**), Kp,uu value in mice (0.23), *in vitro* data (**Figure 1**), and exclusion as a substrate of P-glycoprotein and BCRP shows enough evidence of GLR2007’s potential efficacy for the possible treatment of GBM to support further investigation of GLR2007 through more research and clinical trials (19).

## Data availability statement

The original contributions presented in the study are included in the article/**Supplementary Material**. Further inquiries can be directed to the corresponding author.

## Ethics statement

Ethical review and approval was not required for the animal study because all animal studies reported conformed to the regulations and guidelines regarding animal care and welfare, including the American Association for Accreditation of Laboratory Animal Care International guidelines as reported in the US National Research Council “Guide for the Use of Laboratory Animals, Eighth Edition” (Washington, DC, USA: The National Academies Press; 2011) and People’s Republic of China Ministry of Science and Technology ‘Regulations for the Administration of Affairs Concerning Experimental Animals’ (1988).

## Author contributions

LY contributed to editing the manuscript. ZY contributed to data analysis, study conduction, and editing the manuscript. YW contributed to interpretation of the study data and editing the manuscript. MM contributed to editing the manuscript. All authors contributed to the article and approved the submitted version.

## Funding

The study was funded by Gan and Lee Pharmaceuticals.

## Acknowledgments

The authors would like to thank Dr Naveen Samuel, Gan and Lee Pharmaceuticals, USA, for critical review, and acknowledge Dr Derah Saward-Arav, integrated medhealth communication (imc), UK, for medical writing support, funded by Gan and Lee Pharmaceuticals.

## Conflict of interest

All authors are employees of Gan and Lee Pharmaceuticals.

The authors declare that this study received funding from Gan and Lee Pharmaceuticals. All authors were involved in the design of the study, collection, analysis, and interpretation of data, and in writing the manuscript.

## Publisher’s note

All claims expressed in this article are solely those of the authors and do not necessarily represent those of their affiliated organizations, or those of the publisher, the editors and the reviewers. Any product that may be evaluated in this article, or claim that may be made by its manufacturer, is not guaranteed or endorsed by the publisher.

## Supplementary material

Supplementary Inhibitive effect of GLR2007 and abemaciclib on the proliferation of tumor cell lines. Description of data: Results of *in vitro* cell proliferation assays conducted with 90 cell lines, investigating the impact of GLR2007 and abemaciclib on tumor cell proliferation.
