# Supplementary material for: Investigational cyclin-dependent kinase 4/6 inhibitor GLR2007 demonstrates activity against isocitrate dehydrogenase wild-type glioblastoma and other solid tumors in mice xenograft models
Source: Front Oncol. 2022 Aug 11;12:915862. doi: 10.3389/fonc.2022.915862 (PMC9403987; doi:10.3389/fonc.2022.915862)
Supplement: Supplementary — Inhibitive effect of GLR2007 and abemaciclib on the proliferation of tumor cell lines. Description of data: Results of in vitro cell proliferation assays conducted with 90 cell lines, investigating the impact of GLR2007 and abemaciclib on tumor cell proliferation. [file Table_1.docx]

**Supplementary Table 1. Inhibitive effect of GLR2007 and abemaciclib on the proliferation of tumor cell lines**

|  |  |  |  |  | **IC_50_ (µM)** | |
| --- | --- | --- | --- | --- | --- | --- |
| **Cell line** | **Organism** | **Tissue** | **Tumor type** | **Source** | **Abemaciclib** | **GLR2007** |
| MCF-10A | *Homo sapiens* | Breast | – | Cobioer | 1.77 | 2.73 |
| DBTRG-05MG | *Homo sapiens* | Brain | Glioblastoma | Cobioer | 0.013 | 0.006 |
| U87/DK | *Homo sapiens* | Brain | Glioblastoma | Cobioer | 0.015 | 0.003 |
| IMR32 | *Homo sapiens* | Brain | Neuroblastoma | Cobioer | 0.025 | 0.021 |
| CHP-212 | *Homo sapiens* | Brain | Neuroblastoma | Cobioer | 0.032 | 0.018 |
| LN229 | *Homo sapiens* | Brain | Glioblastoma | Cobioer | 0.035 | 0.017 |
| MOG-G-UVW | *Homo sapiens* | Brain | Grade III astrocytoma | Cobioer | 0.072 | 0.043 |
| SK-N-SH | *Homo sapiens* | Brain | Neuroblastoma | Cobioer | 0.080 | 0.032 |
| U118-MG | *Homo sapiens* | Brain | Glioblastoma | Cobioer | 0.086 | 0.023 |
| U87-MG | *Homo sapiens* | Brain | Glioblastoma | Cobioer | 0.106 | 0.016 |
| KNS-81 | *Homo sapiens* | Brain | Glioblastoma | Cobioer | 0.106 | 0.033 |
| A172 | *Homo sapiens* | Brain | Glioblastoma | Cobioer | 0.112 | 0.041 |
| SK-N-AS | *Homo sapiens* | Brain | Neuroblastoma | Cobioer | 0.138 | 0.066 |
| U251 | *Homo sapiens* | Brain | Glioblastoma | Cobioer | 0.151 | 0.053 |
| T98G | *Homo sapiens* | Brain | Glioblastoma | Cobioer | 0.168 | 0.110 |
| SW1088 | *Homo sapiens* | Brain | Grade II astrocytoma | Cobioer | 0.471 | 0.143 |
| M059K | *Homo sapiens* | Brain | Glioblastoma | Cobioer | 0.871 | 3.247 |
| Daoy | *Homo sapiens* | Brain | Desmoplastic cerebellar medulloblastoma | Cobioer | 13.483 | 3.964 |
| Glioma 261 | *Mus musculus* | Brain | Murine glioma | Cobioer | 2.520 | 1.968 |
| SK-N-BE(2) | *Homo sapiens* | Brain | Neuroblastoma | Cobioer | 0.133 | 0.130 |
| KP-N-YN | *Homo sapiens* | Neural | Neuroblastoma | Cobioer | 0.827 | 0.657 |
| MCF-7 | *Homo sapiens* | Breast | Breast carcinoma | Cobioer | 0.006 | 0.002 |
| T47D | *Homo sapiens* | Breast | Breast carcinoma | Cobioer | 0.012 | 0.001 |
| MDA-MB-231 | *Homo sapiens* | Breast | Breast carcinoma | Cobioer | 0.048 | 0.044 |
| MDA-MB-468 | *Homo sapiens* | Breast | Breast carcinoma | Cobioer | 1.248 | 1.944 |
| 4T1 | *Mus musculus* | Breast | Breast carcinoma | Cobioer | 4.790 | 1.944 |
| Du4475 | *Homo sapiens* | Breast | Breast carcinoma | Cobioer | 10.000 | 2.042 |
| SW620 | *Homo sapiens* | Colon | Colon carcinoma | Cobioer | 0.010 | 0.010 |
| LoVo | *Homo sapiens* | Colon | Colorectal carcinoma | Cobioer | 0.025 | 0.013 |
| HTC116 | *Homo sapiens* | Colon | Colorectal carcinoma | Tong Pai | 0.076 | 0.038 |
| LS174T | *Homo sapiens* | Colon | Colon carcinoma | Cobioer | 0.096 | 0.160 |
| DLD-2 | *Homo sapiens* | Colon | Colorectal carcinoma | Cobioer | 0.237 | 0.033 |
| HCT15 | *Homo sapiens* | Colon | Colorectal carcinoma | Cobioer | 0.294 | 0.043 |
| HCT8 | *Homo sapiens* | Colon | Colorectal carcinoma | Cobioer | 0.384 | 0.081 |
| Caco-2 | *Homo sapiens* | Colon | Colorectal carcinoma | Cobioer | 0.395 | 0.453 |
| COLO 205 | *Homo sapiens* | Colon | Colon carcinoma | Cobioer | 0.432 | 0.194 |
| SW948 | *Homo sapiens* | Colon | Colon carcinoma | Cobioer | 0.586 | 0.189 |
| HT29 | *Homo sapiens* | Colon | Colon carcinoma | Cobioer | 1.841 | 0.601 |
| Colon-26 | *Mus musculus* | Colon | Colorectal carcinoma | Cobioer | 16.725 | 2.120 |
| Colo320DM | *Homo sapiens* | Colon | Colon carcinoma | Cobioer | NA | 1.138 |
| CT26.WT | *Mus musculus* | Colon | Colorectal carcinoma | Cobioer | NA | 2.023 |
| BEL7405 | *Homo sapiens* | Liver | Hepatocellular carcinoma | Cobioer | 2.142 | 1.147 |
| BNL CL.2 | *Mus musculus* | Liver | Normal | Cobioer | 10.000 | 1.936 |
| BRL | *Rattus norvegicus* | Liver | Hepatocellular carcinoma | Cobioer | NA | 2.123 |
| BRL-3a | *Rattus norvegicus* | Liver | Hepatocellular carcinoma | Cobioer | 10.000 | 1.873 |
| H22 | *Mus musculus* | Liver | Hepatocellular carcinoma | Cobioer | 3.584 | 1.839 |
| HCCC9810 | *Homo sapiens* | Liver | Hepatocellular carcinoma | Cobioer | 0.071 | 0.030 |
| HCCLM3 | *Homo sapiens* | Liver | Hepatocellular carcinoma | Cobioer | 3.100 | 1.843 |
| Hep G2 | *Homo sapiens* | Liver | Hepatocellular carcinoma | Cobioer | 3.200 | 2.552 |
| Hepa1-6 | *Mus musculus* | Liver | Hepatocellular carcinoma | ATCC | 1.267 | 0.526 |
| HL7702 | *Homo sapiens* | Liver | Hepatocellular carcinoma | Cobioer | 1.724 | 1.944 |
| HLE | *Homo sapiens* | Liver | Hepatocellular carcinoma | Cobioer | 0.389 | 0.143 |
| Huh-1 | *Homo sapiens* | Liver | Hepatocellular carcinoma | Cobioer | 0.365 | 0.261 |
| Huh-7 | *Homo sapiens* | Liver | Hepatocellular carcinoma | Cobioer | 0.075 | 0.043 |
| JHH-7 | *Homo sapiens* | Liver | Hepatocellular carcinoma | Cobioer | 1.168 | 0.578 |
| Li-7 | *Homo sapiens* | Liver | Hepatocellular carcinoma | Cobioer | 6.291 | 0.712 |
| MHCC97 | *Homo sapiens* | Liver | Hepatocellular carcinoma | Cobioer | 1.100 | 0.353 |
| MHCC97H | *Homo sapiens* | Liver | Hepatocellular carcinoma | Cobioer | 0.137 | 0.066 |
| MHCC97L | *Homo sapiens* | Liver | Hepatocellular carcinoma | Cobioer | 0.236 | 0.101 |
| PLC/PRF/5 | *Homo sapiens* | Liver | Hepatocellular carcinoma | Cobioer | NA | 0.302 |
| SK-HEP-1 | *Homo sapiens* | Liver | Hepatocellular carcinoma | Cobioer | 0.006 | 0.036 |
| SNU-182 | *Homo sapiens* | Liver | Hepatocellular carcinoma | Cobioer | 13.938 | 2.236 |
| SNU-368 | *Homo sapiens* | Liver | Hepatocellular carcinoma | Cobioer | 0.034 | 0.013 |
| SNU-387 | *Homo sapiens* | Liver | Hepatocellular carcinoma | Cobioer | 0.193 | 0.068 |
| SNU-398 | *Homo sapiens* | Liver | Hepatocellular carcinoma | Cobioer | 0.173 | 0.051 |
| SNU-423 | *Homo sapiens* | Liver | Hepatocellular carcinoma | Cobioer | 0.048 | 0.019 |
| SNU-449 | *Homo sapiens* | Liver | Hepatocellular carcinoma | Cobioer | 0.159 | 0.067 |
| SNU-475 | *Homo sapiens* | Liver | Hepatocellular carcinoma | Cobioer | 0.195 | 0.085 |
| SNU-739 | *Homo sapiens* | Liver | Hepatocellular carcinoma | Cobioer | 0.383 | 0.100 |
| SNU-886 | *Homo sapiens* | Liver | Hepatocellular carcinoma | Cobioer | 0.025 | 0.006 |
| HCC827 | *Homo sapiens* | Lung | Lung carcinoma | Cobioer | 0.016 | 0.015 |
| NCI-H1792 | *Homo sapiens* | Lung | Lung carcinoma | ATCC | 0.023 | 0.003 |
| NCI-H441 | *Homo sapiens* | Lung | Lung carcinoma | Cobioer | 0.040 | 0.007 |
| NCI-H358 | *Homo sapiens* | Lung | NSCLC | ATCC | 0.044 | 0.017 |
| SNU-761 | *Homo sapiens* | Lung | Lung carcinoma | Cobioer | 0.054 | 0.008 |
| NCI-H1838 | *Homo sapiens* | Lung | NSCLC | Cobioer | 0.090 | 0.048 |
| NCI-H1703 | *Homo sapiens* | Lung | NSCLC | Cobioer | 0.093 | 0.003 |
| A549 | *Homo sapiens* | Lung | Lung carcinoma | Cobioer | 0.104 | 0.066 |
| NCI-H1975 | *Homo sapiens* | Lung | NSCLC | Cobioer | 0.112 | 0.010 |
| SK-MES-1 | *Homo sapiens* | Lung | Lung carcinoma | Cobioer | 0.148 | 0.067 |
| NCI-H292 | *Homo sapiens* | Lung | Mucoepidermoid pulmonary carcinoma | Cobioer | 0.149 | 0.073 |
| NCI-H1915 | *Homo sapiens* | Lung | NSCLC | Cobioer | 0.324 | 0.053 |
| NCI-H520 | *Homo sapiens* | Lung | Lung carcinoma | Cobioer | 0.364 | 0.241 |
| PC-9 | *Homo sapiens* | Lung | Lung carcinoma | Cobioer | 0.457 | 0.180 |
| NCI-H1299 | *Homo sapiens* | Lung | NSCLC | Cobioer | 0.463 | 0.564 |
| NCI-H460 | *Homo sapiens* | Lung | Lung carcinoma | Cobioer | 0.654 | 0.235 |
| NCI-H23 | *Homo sapiens* | Lung | NSCLC | Cobioer | 0.904 | 0.243 |
| NCI-H2228 | *Homo sapiens* | Lung | NSCLC | Cobioer | 2.074 | 2.054 |
| NCI-H1155 | *Homo sapiens* | Lung | NSCLC | Cobioer | 2.414 | 1.821 |
| NCI-H1581 | *Homo sapiens* | Lung | NSCLC | Cobioer | 3.231 | 1.116 |
| HLF | *Homo sapiens* | Lung | Epidermoid carcinoma | Cobioer | 4.948 | 2.014 |

ATCC, American Type Culture Collection; IC50, half-maximal inhibitory concentration; NA, not available; NSCLC, non-small cell lung cancer.
